# The Adoption of New Treatment Modalities by Health Professionals and the Relative Weight of Empirical Evidence in Favor of Virtual Reality Exposure Versus Mindfulness in the Treatment of Anxiety Disorders

**DOI:** 10.3389/fnhum.2020.00086

**Published:** 2020-03-25

**Authors:** Kevin Nolet, Giulia Corno, Stéphane Bouchard

**Affiliations:** ^1^Cyberpsychology Laboratory of UQO, Department of Psychoeducation and Psychology, Université du Québec en Outaouais, Gatineau, QC, Canada; ^2^LabPsiTec, Departamento de Personalidad, Evaluación y Tratamientos Psicológicos, Universitat de València, Valencia, Spain

**Keywords:** anxiety disorders, exposure therapy, cognitive behavioral therapy, virtual reality, mindfulness

## Abstract

Anxiety disorders are among the most prevalent mental disorders, and cognitive-behavioral therapy (CBT) with exposure exercises is considered as the gold-standard psychological intervention. New psychotherapeutic modalities have emerged in the last decade and, among them, mindfulness has been rapidly adopted by therapists. The adoption rate is slower for the use of virtual reality (VR) to conduct exposure. The goal of the present position paper is to contrast, for the treatment of anxiety disorders, the weight of empirical evidences supporting the use of exposure in VR with the use of mindfulness-based therapy (MBT). Based on the most recent meta-analyses, we found that CBT with exposure conducted in VR was more thoroughly researched and supported than MBT, receiving validation from roughly twice as many studies with high control (i.e., randomized, active controls with clinical samples). However, this conclusion is nuanced by reviewing gaps in the literature for both therapies. Potential factors influencing clinicians’ choice of treatment and suggestions for future research directions are proposed.

## Introduction

Anxiety disorders are highly prevalent (Bandelow and Michaelis, 2015). They involve dysfunctional information processing from the limbic system. As such, cognitive-behavioral therapy (CBT) is recognized as the treatment of choice (Katzman et al., 2014; Nathan and Gorman, 2015; David et al., 2018). CBT is based on the premise that hyperactivation of the amygdala is maintained by the interplay between environmental, biological, cognitive, and behavioral factors and that psychological interventions lead to changes in information processing of threat-related cues from the limbic system through active cognitive and behavioral changes. Techniques based on exposure and its variations, such as behavioral experiments or response prevention, are considered as the key strategies leading to significant clinical improvement (Craske et al., 2014). However, although CBT with exposure has been considered as the gold-standard psychosocial intervention for treating anxiety disorders (Hofmann and Smits, 2008; Otte, 2011), it is not without limitations.

Exposure is usually combined with other cognitive behavior techniques, including anxiety management and cognitive restructuring of dysfunctional cognitions (Abramowitz et al., 2011). A successful exposure allows the client to learn to tolerate her/his fear and anxiety while developing a new behavioral repertoire rather than relying on threat avoidance, leading to new mental associations in the limbic system with lack of threat and with stronger perceived self-efficacy for managing emotions and previously avoided situations. While exposure-based treatments are listed as one of the evidenced-based treatments (EBT) for anxiety disorders by the Division 12 of the American Psychological Association (Chambless and Ollendick, 2001) and the National Institute for Health and Care Excellence (2014) guidelines, its dissemination is confronted with numerous barriers (Hembree and Cahill, 2007). For example, when surveyed about their practice with patients suffering from anxiety disorders, the practitioners report opting for alternative therapies with less empirical support than CBT with exposure. The negative beliefs about exposure, namely, in terms of its safety and tolerability for the patient, and the impracticability of its implementation have been found predictive of the lack of use among therapists (Pittig et al., 2019).

Several therapeutic alternatives to standard exposure have emerged, notably conducting exposure in virtual reality (VR; Wiederhold and Bouchard, 2014). CBT with exposure conducted in VR (CBT-VRexp) has been developed to counter some of the limitations of *in vivo* exposure. By offering a standardized, controlled, and replicable environment that can elicit emotions for therapeutic purposes, VR is a medium that could be more practical and effective for exposure therapy.

Although extensively studied in the scientific community, the use of CBT-VRexp is not widespread among clinicians who tend to favor interventions from other paradigms, such as mindfulness-based therapy (MBT).

Documenting which forms and which psychotherapy and their variations are adopted by therapists and at which rate is challenging. Not only those data are scarce in the literature, they can also vary between mental disorders and countries, inducing biases that further limit the comparisons. In addition to the difficulty in recruiting a representative sample of therapists using probabilistic techniques, generalizing the results on the therapists’ adoption of various forms of psychotherapy could be problematic. Keeping the above limitations in mind, in a survey of German behavioral therapists working in the healthcare system, exposure was used in only 46.8% of treatments focusing on anxiety disorders (Pittig and Hoyer, 2018). Although not specifically focusing on anxiety disorders, Michalak et al. (2020) found, again in a German sample, that up to 82% of licensed therapists integrate MBT in their clinical practice, most of them (80%) using it at least occasionally (fewer than one out of two sessions). However, only 10% of those used a manualized group-based MBT. In their samples, the therapists preferred to integrate in their treatment plans stand-alone interventions such as body scan, breathing meditation, self-soothing with the five senses, or other informal practices. In a sample of practicing CBTs attending a European clinical conference, with the majority working with anxiety disorders, only 13.67% reported using CBT-VRexp occasionally or frequently with their patients (Lindner et al., 2019).

Informal observations rapidly show that the number of training, workshops, and classes on MBT clearly outweigh those on VR or CBT-VRexp. A search on Google^1^ with the keywords “anxiety” and “VR workshops” yielded 230 results and 23,000 results with the keywords “anxiety” and “mindfulness workshops”. The specific numbers vary when other keywords are used, but the ratios remain in the order of 1 to 100 in favor of MBT. Finally, although MBT has experienced a marked increase in scientific and popular interest in the past two decades, recent commentaries (e.g., Farias et al., 2016) have raised questions regarding the evidence base for this family of therapies.

The current paper was motivated by the apparent enthusiasm of mental health professionals to embrace some variations of CBT for anxiety disorders more than others. The aim of this review is to contrast the bulk of evidences supporting the efficacy of treatment of anxiety disorders using CBT-VRexp versus using MBT. The goal is *not* to compare the relative efficacy of both forms of CBT but specifically to compare the amount, or weight, of empirical evidences supporting each of them and contrast it with the therapists’ enthusiasm to adopt each of them. The weight was defined here as the number, and relative efficacy, of randomized controlled trials (RCTs) conducted with clinical samples comparing a treatment with at least another treatment, ideally a treatment considered as an established standard.

## Methods

The general methodology will follow three steps: (a) define CBT-VRexp and MBT, (b) provide a brief overview of their relevance for the treatment of anxiety and its disorders [i.e., as defined in DSM-5, with the addition of obsessive-compulsive disorder and post-traumatic stress disorder (PTSD)], and (c) review and contrast the relative weight of empirical support for both techniques based on already published meta-analyses.

### Defining CBT-VRexp and Mindful-Based Interventions

VR has been defined in different ways, but the practical definition from Schultheis and Rizzo (2001) will be used here: VR is an advanced form of human–computer interface that allows the user to “interact” with and become “immersed” in a computer-generated environment in a naturalistic fashion. Three main features differentiate VR systems from other technologies: immersion, the impression of really being in the environment, and interaction with that environment (e.g., Biocca, 1997; Lombard and Ditton, 1997; Slater, 2009; Fuchs, 2011; Wiederhold and Bouchard, 2014; Cipresso et al., 2018). Computer-generated virtual environments allow clinical assessment, treatment, and rehabilitation, providing interactive ecologically valid scenarios designed to target specific needs.

Cognitive-behavioral therapy-VRexp refers to the use of VR to conduct exposure (Bouchard and Rizzo, 2019). CBT rarely relies only on exposure, although it clearly is the main component of CBT for phobias. Additional therapeutic ingredients include working alliance, case conceptualization, psychoeducation, cognitive reframing, and relapse prevention. For more complex anxiety disorders, the treatment always includes the aforementioned ingredients, plus a stronger involvement of cognitive techniques, and may also involve other techniques, such as problem solving or assertiveness training.

In traditional CBT, many strategies are designed to change internal experiences, such as emotional states (e.g., reducing negative moods), bodily sensations (e.g., reducing pain), and the content of thoughts (e.g., from irrational and/or distorted to rational, realistic, and/or balanced) (Harrington and Pickles, 2009). To the contrary, mindfulness-based approaches teach an alternative way of relating to such experiences. Bishop et al. (2004) identified two basic components of mindfulness: one involves self-regulation of attention and another one involves an orientation toward the present moment in a way characterized by openness, curiosity, and acceptance (Hofmann et al., 2010). In other words, the essential premise underlying mindfulness practices is that experiencing the present moment in a non-judgmental and open way can effectively counter the effects of stressors, as excessive orientation toward the past or future when dealing with stressors can be related to depressive and anxious feelings (e.g., Kabat-Zinn, 2003; Hofmann et al., 2010).

Therefore, mindfulness practice encourages cultivating a new relationship with internal experiences that involves directing attention in a way that it is maintained on immediate experience, without avoiding, over engaging, or elaborating the experience (Kumar et al., 2008). More specifically, it is believed that, by a training focused on approaching stressful situations more reflectively rather than reflexively, mindfulness-based interventions (MBI) can effectively counter the use of avoidance strategies, which attempt to alter the intensity or frequency of unwanted internal experiences (Hayes et al., 2006; Hofmann et al., 2010). These maladaptive strategies are believed to contribute to the maintenance of many, if not all, emotional disorders (Bishop et al., 2004; Hayes, 2004; Hofmann et al., 2010). An important and contrasting feature of MBT is how cognitions are handled. Instead of trying to restructure them, MBT focuses on accepting them and letting them go. However, when it comes to being in contact with feared stimuli, acceptance and orienting attention to fully experience the moment share many similarities with exposure in terms of opportunities to build new mental associations with lack of threat.

### Relevance of VR and MBT in Psychotherapy

VR is used in a wide range of fields, such as physical and neurological rehabilitation (e.g., Schultheis et al., 2002; Holden, 2005; Lam et al., 2006), neuropsychological evaluation (e.g., Rizzo and Buckwalter, 1997; Rizzo et al., 2000), education, and cognitive neuroscience (e.g., Tarr and Warren, 2002). VR started to be used in clinical psychology in the early 1990s. The most common application of VR in clinical psychology has been the treatment of phobias and anxiety-related disorders (i.e., anxiety disorders as defined in the DSM-IV). For example, in the early 1990s, Hodges et al. (1995) reported to have been using virtual environments to provide acrophobic patients with fear-producing experiences of heights in a safe situation. Since that time, VR has been proposed as a new medium for conducting exposure. The rationale behind its use is that the exposure can be conducted with more control from the therapist. CBT-VRExp offers several other advantages over *in vivo* or imaginal exposure (see Côté and Bouchard, 2008 for a detailed list), such as increased attractiveness for patients, more cost-effective, better protection of confidentiality and patient’s safety, etc.

The research in this field shows that VR is able to reduce the anxiety symptoms significantly in different anxiety disorders: social anxiety (SA) disorder (Bouchard et al., 2017), generalized anxiety disorder (GAD) (e.g., Repetto et al., 2013), phobias (e.g., Garcia-Palacios et al., 2002; Parsons and Rizzo, 2008), PTSD (e.g., Gonçalves et al., 2012), panic disorder (PD) and agoraphobia (Botella et al., 2007), and psychological stress (Gaggioli et al., 2014). Studies show that the clinical outcome is superior to waitlist control conditions and comparable to *in vivo* exposure-based interventions. During the last decade, clinicians extended this field to more complex disorders, for instance, eating disorders and body image disturbance (e.g., Ferrer-Garcia et al., 2013; Corno et al., 2018), schizophrenia (e.g., da Costa and de Carvalho, 2004; Freeman, 2008; Kim et al., 2008), and building resilience and post-traumatic growth (e.g., Corno and Bouchard, 2015).

In terms of criticisms, although it is a promising therapeutic medium, adding VR to CBT may not always provide additional benefit to exposure-based therapy (e.g., McLay et al., 2017) and adds costs and complexity to an already effective treatment. The exact role of some psychological factors involved in exposure conducted in VR also needs to be clarified. While the sense of presence, the feeling of being *inside* the virtual environment, has been considered as relevant for treatment success, studies about its actual impact on treatment outcomes have produced mixed results (Botella et al., 2017).

Mindfulness-based therapy has been defined as comprising the third wave of CBT because of its differences with the first two waves, behavior therapy and cognitive therapy (Hayes, 2004; Baer and Sauer, 2009). Having its origins in Eastern Buddhist tradition that is over 2,500 years old, MBT includes mindfulness-based cognitive therapy (MBCT; e.g., Segal et al., 2002) and mindfulness-based stress reduction (MBSR; e.g., Kabat-Zinn, 1982). MBT has become a very popular form of treatment in contemporary psychotherapy (e.g., Kabat-Zinn, 1994; Bishop, 2002; Baer, 2003; Hayes, 2004). For instance, both MBCT and MBSR have demonstrated significant clinical efficacy in the treatment of mood disorders (e.g., Segal et al., 2010), resistant depression (e.g., Kenny and Williams, 2007; Eisendrath et al., 2008), and anxiety disorders (e.g., Evans et al., 2008; Kim et al., 2009; Hofmann et al., 2010). Other clinical applications include pain management, substance use, attention disorders, PTSD, and eating disorders [see Wielgosz et al. (2019) for a review of these applications].

Despite the popularity of MBT, a limited number of clinical trials have specifically examined this treatment in anxiety disorders. More specifically, while the empirical support for the treatment of recurrent depression seems to be strong, the same cannot be as easily said for other clinical-like anxiety disorders. Questions about the methodological qualities of the literature have also been raised, ranging from a lack of active control groups (Farias et al., 2016) to problems in operationalization and measurements (Grossman, 2019).

### Contrasting the Relative Weight of Empirical Support

Previous researchers have worked in great lengths to find all available outcome studies on CBT-VRexp and MBT in order to publish meta-analyses, and contrasting the adoption of treatment modalities by therapists based on information already available to them leads to a fairer comparison. Therefore, our source of information to balance the weight of evidences providing empirical support for both techniques is based on the most recent and comprehensive meta-analyses for each treatment modality. We searched the Scopus database for meta-analyses on clinical trials for MBT, CBT-VRexp, and also CBT with *in vivo* exposure as a gold-standard comparison. We complemented this search with Google Scholar to ensure that all relevant papers were found. We used the following terms in the title, abstract, and keywords: “meta-analysis” and “anxiety”, combined with “mindfulness”, “virtual reality” or “VR” or “VRET”, and “CBT” or “exposure therapy” or “cognitive behavioral therapy”. We limited our search to papers written in English and published in peer-reviewed journals. To ensure the longest possible coverage, we aimed for the most recent meta-analysis, thus limiting our search to papers published between 2018 and July 15, 2019. The papers were reviewed by the first author following specific inclusion and exclusion criteria.

The following criteria were used to identify the meta-analyses that responded to our needs: (1) the longest coverage possible (i.e., the most recent papers going as far back possible in publication history), (2) information available on the randomization procedure used for the studies included, (3) effect sizes (ES) for each control type separately (i.e., inactive, active, and evidence-based), (4) ES calculated on an anxiety measure, and (5) preferably with patients diagnosed with an anxiety- or stress-related disorder or with a score above the cutoff on a clinical measure. When available, we also used results pertaining to attrition and deterioration rates within these meta-analyses.

To make sure that the meta-analyses included were representative of a larger part of the literature, we excluded papers if (1) they were limited to a specific population (e.g., youth or elderly), (2) they were limited to one particular therapy or modality (e.g., self-compassion for MBT and online interventions), and (3) they were limited to one diagnostic category (e.g., phobias for CBT-VRexp) or did not provide information about each included category individually; to make sure that the ES were observed specifically for anxiety, we excluded meta-analyses if (4) they aggregated heterogenous outcomes (e.g., psychological distress); and papers were also excluded if (5) the treatment modality was not objectively isolated (e.g., by adding a new modality to the basic treatment; see Supplementary Material for a complete list of the papers reviewed).

## Results

For MBT, the search yielded 76 hits. An initial screening eliminated 39 papers because they were limited in their population scope (e.g., children, youth, and cancer survivors), 15 were not meta-analyses, seven were not about anxiety disorders, three were not about MBT, two were limited to online interventions, and four were limited to self-compassion, self-help, or stand-alone interventions. Of the remaining six papers, two were further discarded because they agglomerated heterogenous outcomes (e.g., “internalizing symptoms”, de Abreu Costa et al., 2019; “negative affectivity”, Schumer et al., 2018), two did not report ES separately for each intervention and/or each control category (Bandelow et al., 2018; Hedman-Lagerlof et al., 2018), and one which had raised many methodological concerns^2^ (Singh and Gorey, 2018). Thus, the meta-analysis from Goldberg et al. (2018) was retained for our study (see Supplementary Material 1).

For CBT-VRexp, the search yielded 12 hits. Three papers were removed because they were not meta-analyses, three were not about CBT-VRexp or specific to this intervention, one was not specific to anxiety disorders, and one was limited in population scope (children). Of the four remaining papers, the meta-analysis from Carl et al. (2019) was retained for our study, supplemented with the one by Benbow and Anderson (2019) for attrition data. The remaining two publications were rejected because they were either limited to a specific diagnostic category (SA; Chesham et al., 2018) or focusing on deterioration data (Fernández-Álvarez et al., 2019) (see Supplementary Material 2).

For CBT, a total of 96 hits were obtained from our search. Of these, eight papers were rejected because they were not about CBT, 11 were not meta-analyses, 25 were not about anxiety disorders, 31 were limited in their population scope, two were not about treatment efficacy, five were limited to Internet or computer-based intervention, and six were about CBT-VRexp or MBT. Of the eight remaining papers, one was rejected because it was limited in scope (group therapy for PTSD; Schwartze et al., 2019), one was too restrictive on the measure of outcome to allow comparisons (remission rate; Springer et al., 2018), two studied the effect of added interventions to CBT (Bernard et al., 2018; Marker and Norton, 2018), and two were limited to primary care settings without information about specific anxiety disorders (Zhang et al., 2019a, b). Since Barry et al. (2018) did not provide information about RCTs, we favored Carpenter et al. (2018) for our study (see Supplementary Material 3).

A summary of the information extracted from published meta-analyses documenting the efficacy of CBT-VRExp and MBT is reported in Table 1, with the meta-analysis comparing CBT with *in vivo* exposure to other active control treatments reported as a reference for comparison. For manualized MBT, Goldberg et al. (2018) identified 22 RCTs with clinical samples of anxiety disorders and PTSD, totaling 26 ES. Of those, nine used an active control and only seven compared the efficacy over an EBT, such as CBT with *in vivo* exposure. In comparison, of the 30 RCT studies identified for CBT-VRexp (Carl et al., 2019) totaling 40 ES, twice as many (14) used CBT with *in vivo* exposure as a control intervention.

**TABLE 1:** Documenting the relative weight of evidences, based on the number of clinical trials on MBT and CBT-VRexp and on *in vivo* exposure as a reference, and the pooled effect sizes.

	**MBT^1^**	**CBT-VRexp^2,3^**	**CBT-*in vivo* exp^4^**
Number of published randomized controlled trials	22	30	>40
Total number of ES	26	40	>40
Number of ES with clinical samples	26	39	>40
Number of ES with an inactive control at post-treatment	10	20	N/A
Number ES with an active control at post-treatment	9	6	40
Pooled ES for comparisons with active controls at post-treatment	0.27 (0.06, 0.67)	0.78 (0.25, 1.31)	0.56 (0.44, 0.69)
Number of ES with *in vivo* exposure	7	14	N/A
Pooled ES for comparisons with inactive controls at the follow-up	−0.07 (−0.63, 0.48)	0.90 (−0.05, 1.85)	N/A
Number of ES with a follow-up	9	11	>22
Attrition rates	17%	16%	24%

*Data are based on meta-analyses on anxiety-related disorders. CBT, cognitive-behavioral therapy; EBT evidence-based treatment; *in vivo* exp, real-life exposure; ES, effect sizes; MBT, mindfulness-based therapy; VRexp, virtual reality-based exposure. Goldberg et al. (2018) did not pool PTSD studies with those of anxiety disorders in their analysis, contrary to the other meta-analyses presented here. After contacting the main author, who accepted to share his database, we performed a re-analysis of the data (Mantel–Haenszel odds ratio) with the PTSD and the anxiety disorder studies pooled together. Sources of information: ^1^Goldberg et al., 2018. ^2^Carl et al., 2019 for all data except for attrition rates. ^3^Benbow and Anderson, 2019 for attrition rates. ^4^Carpenter et al., 2018 for a meta-analysis including only comparisons with active controls.*

The nature of clinical samples also differs between the MBT and the CBT-VRexp studies retained in the various meta-analyses. For MBT, eight studies used SA samples, seven for PTSD, five for GAD, 2 for obsessive-compulsive disorder, and three mixed samples. For CBT-VRexp, the bulk of evidence pertains to specific phobias (SP) with 17 studies, followed by 13 for SA. Less frequent evidence was documented for PTSD and PD, with five studies each.

The number of clinical trials reporting follow-up data in the meta-analyses is much smaller and not very different between MBT and CBT-VRexp. For MBT, only two studies fitting the criteria of the meta-analyses documented the long-term efficacy compared to an EBT (CBT with *in vivo* exposure), compared to seven for CBT-VRexp. The average effect size of the comparisons with inactive or active control conditions was consistently lower in MBT compared to that in CBT-VRexp. The attrition rate reported in studies on MBT and CBT-VRexp was very similar. Odds of dropping out of MBT and CBT-VRexp were not different from other EBT.

Overall, the amount of information documenting the efficacy of using CBT-VRexp for anxiety disorders is about twice as much as for MBT. Note that our analysis is about the relative number of evidences; the comparative efficacy has not yet been empirically tested and comparing the pooled ES in Table 1 may be hazardous. Nevertheless, these observations about available evidence from the published literature cannot justify the disproportionately larger acceptance and enthusiasm of MBT over CBT-VRexp.

## Discussion

Although CBT with exposure exercises has been considered as the gold-standard treatment for anxiety disorders, researchers and clinicians in mental health have embraced and combined different approaches to overcome some limits of CBT and exposure. In this article, we focused on two forms of CBT, CBT-VRexp, and MBT. Specifically, this study was driven by the wish to document and reflect on the apparent widespread scientific and popular interest and preference in using MBT over the use of CBT-VRexp in the treatment of anxiety disorders. Therefore, the aim of this study was to contrast the bulk of evidences supporting the efficacy, specifically for the treatment of anxiety disorders, of using CBT-VRexp versus using MBT. Faced with the growing hype around mindfulness, among both the general population and the clinicians, our question was: is this hype empirically supported? Reviewing studies gathered in meta-analyses, we found twice as many studies supporting CBT-VRexp over MBI. When looking at comparisons with CBT plus *in vivo* exposure, twice as many studies were published in support of CBT-VRexp (14) compared to MBT (seven). Strength is in the numbers: with more studies, the pooled ES are more robust and less likely to be artificially inflated by publication bias. We can also note that these ES are higher in favor of CBT-VRexp compared to MBT. The available information in meta-analyses reported large pooled ES favoring CBT-VRexp over active control conditions at post-treatment and over inactive control conditions (e.g., waiting list or no treatment) at follow-up. However, the pooled ES favoring MBT over active controls were small at post-treatment and even smaller when compared with inactive controls at follow-up. Overall, while expected changes are still clinically significant for MBT, stronger effects with more empirical support are found for CBT-VRexp for treating anxiety disorders.

However, nuances exist when looking at specific diagnostic categories. SA is by far the most studied diagnosis, both for CBT-VRexp and MBT, with respectively six and four published RCT against EBT^3^ in the meta-analyses that we used. For PD and SP, only papers for CBT-VRexp were found. For GAD and PTSD, the studies included in the meta-analyses were only for MBT. Thus, based on the evidences, the relevance of MBT or CBT-VRexp was also carefully considered given the target disorder.

In all cases, the number of studies with follow-up data is low, both in terms of numbers and duration. Not enough data are available to draw conclusions about specific disorders. Nonetheless, clinicians may choose CBT-VRexp over MBT with some confidence for its long-term effect as more studies in the meta-analyses found no difference in the long run with the participants treated with EBT.

Another important limitation in the literature that practitioners should keep in mind is that deterioration data are rarely reported for both CBT-VRexp and MBT. This is not surprising as it is a relatively new line of inquiry for clinical efficacy studies. It is also challenging as it requires monitoring of individual data compared to group-level analysis. Yet more papers in the literature report such data for CBT-VRexp (around 40%; Fernández-Álvarez et al., 2019) compared to MBT (around 15%; Wong et al., 2018). Deterioration rates reported in the literature were lower for both patients receiving CBT-VRexp (4.0%) and other forms of treatment (2.8%) compared to the wait-listed control (15%; Fernández-Álvarez et al., 2019), while deterioration was reported for only 1% of the participants in both the MBT and the control groups [although only three studies included samples with an anxiety disorder or PTSD (Wong et al., 2018)]. While practicing meditation could be thought of as relatively harmless, this might not be the case in patients suffering from a diagnosed mental disorder. Also, mindfulness practice can be unpleasant and challenging without causing harm. As suggested by Baer et al. (2019), systematic research is needed to address this question, which would require monitoring individual data like what Fernández-Álvarez et al. (2019) did in their analysis. While more studies reported such data for CBT-VRexp, there is still room for improvement. Adverse effects can come in many forms that are not constantly measured (Fernández-Álvarez et al., 2019), such as cybersickness symptoms (i.e., feelings of nausea, dizziness, and discomfort) when using VR technology. While these symptoms do not deteriorate the condition of the patients, they might hinder their capacity to profit from the intervention.

The results of the present study lead to an interesting question: given the higher frequency of support found for CBT-VRexp over MBT for treating anxiety disorders, why has MBT attracted a much wider scientific and popular interest compared to CBT-VRexp? We have formulated some tentative answers.

One possible answer to our question could be the cost and apparent complexity of using VR technologies. While high-end technologies are costly and thus more suitable for research purposes, head-mounted display systems are increasingly suitable for the general public. Indeed 3 years ago affordable headsets became available, and now the technology is becoming even more affordable and more immersive^4^. Yet in order to use VR, researchers and clinicians need virtual environments (software) and some hardware, which may be seen as cumbersome and represent additional costs. Thus, applying MBT, which “only” requires training from the health professional, could be seen as more affordable and more attractive than using technologies.

A likely answer could also be that, unfortunately, some professionals do not rely on empirical data to choose their therapeutic interventions but rather on their preferences, the appeal of the model, and the current trends in clinical orientations. Adopting an intuitive thinking style is predictive of more negative attitudes toward EBT requiring exposure and more positive ones toward the adoption of alternative therapeutic interventions (Gaudiano et al., 2011). MBT is part of the current zeitgeist (Michalak and Heidenreich, 2018): psychological stress and its reduction are major concerns in modern societies, and MBT offers a solution appealing for both scientific and spiritual reasons. At the same time, government agencies start to adopt MBT as a first-line treatment for anxiety and depression, such as the National Health Service in the United Kingdom (Mindfulness All Party Parliamentary Group [MAPPG], 2015). Thus, both psychological and sociological factors would influence clinicians in their adoption of MBT in their practice. CBT-VRexp might not have the same appeal.

There are still worries over the use of CBT-VRexp. One which is frequently cited is that VR technology could hinder the therapeutic relationship. This is not the case, as shown by Ngai et al. (2015). In addition, therapists from Lindner et al. (2019) rated “making exposure less stressful” as an advantage of using VR and “patients experiencing the VR environment as too real” as a disadvantage. Endorsements of these items by therapists might be indicative of a misunderstanding of exposure and a reluctance to induce anxiety in one’s patients, even for their own benefit. This could lead to the adoption of alternative clinical interventions, such as MBT, not because it would benefit the patient but because it is easier on the therapist. In fact, novice therapists tend to have comparable stress levels to their patients, both subjectively and physiologically, right before engaging in exposure compared to a control therapy session (Schumacher et al., 2014). Also, novice therapists experimentally led by researchers to hold negative beliefs about exposure did deliver the treatment sub-optimally by being overly cautious compared to those in the positive beliefs condition (Farrell et al., 2013). Many therapists deliver exposure at lower doses (i.e., for a shorter period of time and in less threatening situation) and in conjunction with controlled breathing strategies without clear empirical evidence of its added clinical efficacy (Deacon et al., 2013). In sum, CBT-VRexp may be less attractive essentially because it is a form of exposure, and therapists may not like to do exposure with their patients. Achieving suboptimal results with their patient could reinforce their negative beliefs about exposure and further justify the choice of alternative interventions. Finally, if therapists avoid using exposure because of fear of patients’ discomfort, they may also favor MBT in ways that reduce its usefulness to develop new associations with lack of threat and that reinforce avoidance. When facing the avoided stimuli, MBT can be used constructively to foster exposure (e.g., “Let us be fully aware that there is a live spider crawling on the table, that it is disgusting and looking at you, and embrace the situation and how you can remain in it”) or less constructively to foster avoidance (e.g., “Although there is a spider here, let us focus on your respiration and your body, let go of your worries about the spider, and pace your breathing to slowly calm down”). Doing mindful breathing exercises in conjunction with CBT-VRexp could diminish its effectiveness by reducing the anxiety of the patient, thus acting as a form of avoidance. Exposure requires the patient to experience the threatening stimuli to learn through experience that it is safe. To do so, the therapists must tolerate the idea of being “responsible” of “inflicting” anxiety to their patients, which can prompt them to seek out alternative interventions or to supplement them with tempering ones. There is a thin line that is very easy to cross in favor of helping patients develop avoidance behaviors that will either be detrimental in the long term or will need to always be used by patients as safety seeking behaviors or neutralization in order to cope.

### Future Directions

First, researchers should begin gathering data on the fit between patients and treatment modalities. While efficacy studies are important, their focus is at the group level. While we can show that two treatments are equally effective in the treatment of a disorder for two randomly selected individuals, nothing tells us that they will respond equally well to both treatments. For example, some people have a hard time feeling immersed in a VE (e.g., they have cybersickness symptoms), thus not responding to the stimuli used in the exposure therapy. Others do not adhere to the mindfulness philosophy and will not meditate or practice acceptance at home. To better inform professionals about choosing therapeutic approaches, future research should include measures of potential predictors of treatment efficacy. With such information, clinicians could take the best decision for their patient based not only on what science tells them that is effective but also on why it works that way for some people and not for others.

To date, research on CBT-VRexp has mostly been about replicating *in virtuo* what can normally be done *in vivo*. Doing so, researchers were able to demonstrate that VR is useful to do exposure with hard-to-access stimuli in a controlled and secure environment. Thus, their line of scientific inquiry was mostly focused on phobias. As a result, VR might have been less attractive to professionals. The field of VR is now addressing the more complex anxiety disorders to provide solutions for patients that may be more frequent in therapists’ caseloads (e.g., OCD, PTSD, GAD). Still we feel that this is a missed opportunity. VR could be used to improve exposure therapy by pushing its limits way further than what can be done *in vivo*, thus allowing to build stronger associations with lack of threat than what can be done *in vivo*. For example, VR is not limited to visual and auditory stimuli. Studies have successfully integrated olfactory (Baus and Bouchard, 2014), haptic (Tremblay et al., 2016), and thermal (Shaw et al., 2019) stimuli to VE. This could potentially lead to a multisensory exposure therapy for PTSD, bringing recollection of the traumatic experience and reprocessing to a new level. VR can also be used to expose patients to situations hard or impossible to do *in vivo*. For acrophobia, a therapist using CBT-VRexp could ask his/her patient to dance on the edge of a virtual cliff, test his/her balance, and actually jump at will over the cliff to confront his/her fear of falling. For social phobia, it is possible in VR to ask people on dates or set up social blunders that would be delicate to do with actual people.

Stand-alone MBI, as opposed to full MBT treatment programs, are of special note here as they are the most commonly reported form of mindfulness intervention used by clinicians (Michalak et al., 2020). This is not surprising and probably not limited to MBT, with less than 2% of psychotherapists reporting adopting only one practice orientation (Cook et al., 2010). For our review on studies with anxiety disorders, our comparisons were with studies using some form of MBI. The meta-analysis from Blanck et al. (2018), which was not used in our study because it had less RCT than that of Goldberg et al. (2018), focused on studies where only MBI were used as a stand-alone treatment. Of the 21 studies identified by Blanck et al. (2018), only five were RCT. None compared the efficacy of MBI as a stand-alone treatment to an EBT and, most importantly, none used clinical samples. No data were available for long-term effect nor adverse effects. Moreover, no study tested the impact of integrating MBI to other validated intervention protocols. This lack of empirical support could be problematic if the intervention in the integrated stand-alone treatment does not fully address the therapeutic goals of a full MBT program and does not include adequate exposure strategies. The question remains: does the professional choose a treatment strategy to avoid discomfort in their patients? Given that the therapist’s experiential avoidance is a significant negative predictor of choosing exposure as a treatment option (Scherr et al., 2015), the question deserves an empirical answer.

Among the limits of the current paper, the first that comes to mind is the reliance on meta-analyses. Meta-analyses are imprecise and limited by design. Variations in inclusion criteria, search terms, and search engines can yield important differences in the result. Given the publication process, papers published in 2018 or 2019 has a coverage running up to 2017, thus important articles could have been left out of this review. The goal of this review was not to be exhaustive but to contrast the state of the literature. It would be quite surprising if the gap in the number of RCT between MBT and CBT-VRexp had been filled in the last year. Conducting our own systematic search of the literature, including unpublished reports and theses, may have provided with more precise numbers, but the ratio of evidences would have remained in the same range.

Another problem of reviewing and comparing meta-analyses is that we had no control on how the results were reported, namely, which parameters were used. For example, we were unable to report on the methodological quality evaluation of the studies as different indexes were used across meta-analyses or simply not reported. Most biases toward CBT-VRexp and MBT are the same and those found in the literature on clinical efficacy: selective reporting, small sample sizes, no intent-to-threat analyses, no deterioration analyses, or adverse effects reporting.

## Conclusion

The objective of this paper was to contrast, specifically for the treatment of anxiety disorders, the weight of evidences supporting the use of exposure in VR versus the use of MBT. Overall, the results of the comparisons have shown that CBT with exposure conducted in VR was more thoroughly researched and supported than MBT. Nevertheless, this conclusion is nuanced by reviewing several gaps in the literature for both therapies, and much more research is required to establish which therapies for the treatment of anxiety disorders are suitable, how they should be carried out, and for whom.

## Author Contributions

KN, GC, and SB designed the study. KN and GC collected and analyzed the data and drafted the manuscript. SB facilitated the study execution and aided in the interpretation of findings. All the authors critically reviewed the draft and made significant contributions to the final version.

## Conflict of Interest

SB is the President of, and own equity in, Cliniques et Développement In Virtuo, a spin-off company from the university that uses virtual reality and distributes virtual environments designed for the treatment of mental disorders. The terms of these arrangements have been reviewed and approved by the Université du Quebec en Outaouais in accordance with its conflict of interest policies. The remaining authors declare that the research was conducted in the absence of any commercial or financial relationships that could be construed as a potential conflict of interest.

## Footnotes

1 On July 5, 2019.

2 Singh and Gorey (2018) paper is of special note. It directly addresses our subject matter, yet we have doubts on the robustness of their methodology. While they reported 9 RCT studies directly comparing MBI and CBT, they included 2 studies with the same sample, 2 studies on MBI with added exposure, 1 with CBT as a control group without clear definition (TAU with or without medication, not on the whole sample) and with depressive and/or anxious participants (again without stats on each diagnostic category included), 1 study with a 1-h intervention, and 1 with an error on the ES reported (advantage for MBI that was originally found for CBT). This leaves a total of 3 valid studies, which is more in line with Goldberg et al. (2018) and Hedman-Lagerlof et al. (2018). For these reasons, with opted to exclude this paper and report results from the former.

3 Pooled effect size for MBT was recalculated from Goldberg et al. (2018) with SA studies only: MBT was found to be significantly less effective than EBT, −0.31 (−0.60, −0.03).

4 Retrieved from: https://www.wired.com/story/oculus-rift-s-vr-headset/ and http://time.com/4169430/oculus-rift-price-release-date-2016/
