# Supplementary material for: The Adoption of New Treatment Modalities by Health Professionals and the Relative Weight of Empirical Evidence in Favor of Virtual Reality Exposure Versus Mindfulness in the Treatment of Anxiety Disorders
Source: Front Hum Neurosci. 2020 Mar 25;14:86. doi: 10.3389/fnhum.2020.00086 (PMC7109262; doi:10.3389/fnhum.2020.00086)
Supplement: Supplementary file 1 [file Data_Sheet_1.docx]

Supplementary Material

**1 Supplemental material 1: Excluded studies on Mindfulness-based interventions (MBI)**

| **Study** | **Main reason for exclusion** |
| --- | --- |
| Andrasik et al., 2018 | Limited population scope |
| Bamber and Morpeth, 2019 | Limited population scope |
| Bandelow et al., 2018 | No ES separately for each intervention and/or each control category |
| Bartlett et al., 2019 | Limited population scope |
| Blanck et al., 2018 | Limited intervention modality |
| Borquist-Conlon et al., 2019 | Limited population scope |
| Breedvelt et al., 2019 | Limited population scope |
| Bulzacka et al., 2018 | Not a meta-analysis |
| Cavicchioli et al., 2018 | Not for anxiety disorders |
| Chu et al., 2018 | Not for anxiety disorders |
| Dasnair et al., 2018 | Limited intervention modality |
| de Abreu Costa et al., 2019 | Aggregation of heterogeneous outcomes |
| Dunning et al., 2019 | Limited population scope |
| Ewais et al., 2019 | Limited population scope |
| Fangtham et al., 2019 | Limited population scope |
| Ferrari et al., 2019 | Limited intervention modality |
| Fulton et al., 2018 | Limited population scope |
| Gallagher and Feder, 2018 | Not a meta-analysis |
| Ghielen et al., 2019 | Limited population scope |
| Graser and Stangier, 2018 | Not a meta-analysis |
| Halladay et al., 2019 | Limited population scope |
| Hedman-Lagerlof et al., 2018 | No ES separately for each intervention and/or each control category |
| Huang et al., 2018 | Limited population scope |
| Huguet et al., 2018 | Not about MBI |
| Janssen et al., 2018 | Limited population scope |
| Kirby and Gilbert, 2019 | Not a meta-analysis |
| Laird et al., 2018 | Not a meta-analysis |
| Levy et al., 2018 | Not a meta-analysis |
| Li and Bressington, 2019 | Limited population scope |
| Liu et al., 2018 | Limited population scope |
| Lo et al., 2018 | Limited population scope |
| Lomas et al., 2018 | Limited population scope |
| Lomas et al. 2019 | Limited population scope |
| López-Pinar et al., 2019 | Limited population scope |
| Loughnan et al., 2018 | Limited population scope |
| Lovas and Schuman-Olivier, 2018 | Not for anxiety disorders |
| Mahoney et al., 2019 | Limited population scope |
| McClintock et al., 2019 | Not about MBI |
| Mehta, 2018 | Not a meta-analysis |
| Meister and Juckel, 2018 | Not for anxiety disorders |
| Mendelson and Eaton, 2018 | Not a meta-analysis |
| Michaelis et al., 2018 | Limited population scope |
| Modica and Hoenig, 2018 | Not a meta-analysis |
| Niknejad et al., 2018 | Limited population scope |
| O'Connor et al., 2018 | Limited intervention modality |
| Perach et al., 2019 | Limited population scope |
| Perich and Mitchell, 2019 | Limited population scope |
| Petrie et al., 2019 | Limited population scope |
| Prothero et al., 2018 | Not for anxiety disorders |
| Robins, 2019 | Not a meta-analysis |
| Ruffault et al., 2019 | Not a meta-analysis |
| Saeed et al., 2019 | Not a meta-analysis |
| Schell et al., 2019 | Limited population scope |
| Schumer et al. 2018 | Aggregation of heterogeneous outcomes |
| Scott-Sheldon et al., 2019 | Limited population scope |
| Selchen et al., 2018 | Not a meta-analysis |
| Sesel et al., 2019 | Limited population scope |
| Sesel et al., 2018 | Limited population scope |
| Sevilla-Llewellyn-Jones et al., 2018 | Limited intervention modality |
| Simpson et al., 2019 | Limited population scope |
| Singh and Gorey, 2018 | Multiple methodological problems (see footnote 2 in main article) |
| Slimani et al., 2018 | Not for anxiety disorders |
| Smith et al., 2019 | Limited population scope |
| Spinelli et al., 2019 | Limited population scope |
| Temple et al., 2018 | Limited population scope |
| Tian et al., 2019 | Limited population scope |
| Vakonaki et al., 2018 | Not for anxiety disorder |
| Vasudev et al., 2019 | Not a meta-analysis |
| Wilson et al., 2019 | Limited intervention modality |
| Witt et al., 2019 | Limited population scope |
| Yusufov et al., 2019 | Limited population scope |
| Zhang et al., 2019 | Limited population scope |
| Zill et al., 2018 | Limited population scope |
| Zou et al., 2018 | Not about MBI |

**References**

Andrasik, F., Grazzi, L., Sansone, E., D'Amico, D., Raggi, A., and Grignani, E. (2018). Non -pharmacological approaches for headaches in young age: An updated review. *Front. Neurol*. 9. doi:10.3389/fneur.2018.01009

Bamber, M. D., and Morpeth, E. (2019). Effects of Mindfulness Meditation on College Student Anxiety: a Meta-Analysis. *Mindfulness*. 10, 203-214. doi:10.1007/s12671-018-0965-5

Bandelow, B., Sagebiel, A., Belz, M., Görlich, Y., Michaelis, S., and Wedekind, D. (2018). Enduring effects of psychological treatments for anxiety disorders: Meta-Analysis of follow-up studies. *Br. J. Psychiatry.* 212, 333-338. doi:10.1192/bjp.2018.49

Bartlett, L., Martin, A., Neil, A. L., Memish, K., Otahal, P., Kilpatrick, M., et al. (2019). A systematic review and meta-analysis of workplace mindfulness training randomized controlled trials.  *J. Occup. Health Psychol*. 24, 108-126. doi:10.1037/ocp0000146

Blanck, P., Perleth, S., Heidenreich, T., Kröger, P., Ditzen, B., Bents, H., et al. (2018). Effects of mindfulness exercises as stand-alone intervention on symptoms of anxiety and depression: Systematic review and meta-analysis. *Behav. Res. The.* 102, 25-35. doi:10.1016/j.brat.2017.12.002

Borquist-Conlon, D. S., Maynard, B. R., Brendel, K. E., and Farina, A. S. J. (2019). Mindfulness-Based Interventions for Youth With Anxiety: A Systematic Review and Meta-Analysis. *Res. Soc. Work Pract.* 29, 195-205. doi:10.1177/1049731516684961

Breedvelt, J. J. F., Amanvermez, Y., Harrer, M., Karyotaki, E., Gilbody, S., Bockting, C. L. H., et al. (2019). The effects of meditation, yoga, and mindfulness on depression, anxiety, and stress in tertiary education students: A meta-analysis. *Front. Psychiatry*. 10, 193. doi:10.3389/fpsyt.2019.00193

Bulzacka, E., Lavault, S., Pelissolo, A., and Bagnis Isnard, C. (2018). Mindful neuropsychology: Mindfulness-based cognitive remediation. *Encephale*. 44, 75-82. doi:10.1016/j.encep.2017.03.006

Cavicchioli, M., Movalli, M., and Maffei, C. (2018). The Clinical Efficacy of Mindfulness-Based Treatments for Alcohol and Drugs Use Disorders: A Meta-Analytic Review of Randomized and Nonrandomized Controlled Trials. *Eur. Addict.* *Res*. 24, 137-162. doi:10.1159/000490762

Chu, C. S., Stubbs, B., Chen, T. Y., Tang, C. H., Li, D. J., Yang, W. C., et al. (2018). The effectiveness of adjunct mindfulness-based intervention in treatment of bipolar disorder: A systematic review and meta-analysis. *J. Affect. Disord*. 225, 234-245. doi:10.1016/j.jad.2017.08.025

Dasnair, R., Martin, K. J., and Golijani-Moghaddam, N. (2018). Mindfulness self-help interventions for symptoms of depression, anxiety and stress: Review and meta-analysis. *Int. J. The.r Rehabil.* 25, 82-95. doi:10.12968/ijtr.2018.25.2.82

de Abreu Costa, M., D’Alò de Oliveira, G. S., Tatton-Ramos, T., Manfro, G. G., and Salum, G. A. (2019). Anxiety and Stress-Related Disorders and Mindfulness-Based Interventions: a Systematic Review and Multilevel Meta-analysis and Meta-Regression of Multiple Outcomes. *Mindfulness.* 10, 996-1005. doi:10.1007/s12671-018-1058-1

Dunning, D. L., Griffiths, K., Kuyken, W., Crane, C., Foulkes, L., Parker, J., et al. (2019). Research Review: The effects of mindfulness-based interventions on cognition and mental health in children and adolescents – a meta-analysis of randomized controlled trials. *J. Child. Psychol. Psychiatry.* 60, 244-258. doi:10.1111/jcpp.12980

Ewais, T., Begun, J., Kenny, M., Rickett, K., Hay, K., Ajilchi, B., et al. (2019). A systematic review and meta-analysis of mindfulness based interventions and yoga in inflammatory bowel disease. *J. Psychosom. Res.* 116, 44-53. doi:10.1016/j.jpsychores.2018.11.010

Fangtham, M., Kasturi, S., Bannuru, R. R., Nash, J. L., and Wang, C. (2019). Non-pharmacologic therapies for systemic lupus erythematosus. *Lupus.* 28, 703-712. doi:10.1177/0961203319841435

Ferrari, M., Hunt, C., Harrysunker, A., Abbott, M. J., Beath, A. P., and Einstein, D. A. (2019). Self-Compassion Interventions and Psychosocial Outcomes: a Meta-Analysis of RCTs. *Mindfulness*. 8, 1455-1473. doi:10.1007/s12671-019-01134-6

Fulton, J. J., Newins, A. R., Porter, L. S., and Ramos, K. (2018). Psychotherapy targeting depression and anxiety for use in palliative care: A meta-analysis. *J. Palliat. Med*. 21, 1024-1037. doi:10.1089/jpm.2017.0576

Gallagher, R., and Feder, M. A. (2018). Adult ADHD: Psychosocial treatment components and efficacy status. *Psychiat. Ann.* 48, 333-337. doi:10.3928/00485713-20180606-02

Ghielen, I., Rutten, S., Boeschoten, R. E., Houniet-de Gier, M., van Wegen, E. E. H., van den Heuvel, O. A., et al. (2019). The effects of cognitive behavioral and mindfulness-based therapies on psychological distress in patients with multiple sclerosis, Parkinson's disease and Huntington's disease: Two meta-analyses. *J. Psychosom. Res.* 122, 43-51. doi:10.1016/j.jpsychores.2019.05.001

Graser, J., and Stangier, U. (2018). Compassion and Loving-Kindness Meditation: An Overview and Prospects for the Application in Clinical Samples. *Harv. Rev. Psychiatry.* 26, 201-215. doi:10.1097/HRP.0000000000000192

Halladay, J. E., Dawdy, J. L., McNamara, I. F., Chen, A. J., Vitoroulis, I., McInnes, N., et al. (2019). Mindfulness for the Mental Health and Well-Being of Post-Secondary Students: A Systematic Review and Meta-Analysis. *Mindfulness.* 10, 397-414. doi:10.1007/s12671-018-0979-z

Hedman-Lagerlof, M., Hedman-Lagerlof, E., and Ost, L. G. (2018). The empirical support for mindfulness-based interventions for common psychiatric disorders: a systematic review and meta-analysis. *Psychol. Med*. 48, 2116-2129. doi:10.1017/S0033291718000259

Huang, J., Nigatu, Y. T., Smail-Crevier, R., Zhang, X., and Wang, J. (2018). Interventions for common mental health problems among university and college students: A systematic review and meta-analysis of randomized controlled trials. *J. Psychiat. Res. 107*, 1-10. doi:10.1016/j.jpsychires.2018.09.018

Huguet, A., Miller, A., Kisely, S., Rao, S., Saadat, N., and McGrath, P. J. (2018). A systematic review and meta-analysis on the efficacy of Internet-delivered behavioral activation. *J. Affect. Disord.* 235*,* 27-38. doi:10.1016/j.jad.2018.02.073

Janssen, M., Heerkens, Y., Kuijer, W., Van Der Heijden, B., and Engels, J. (2018). Effects of mindfulness-based stress reduction on employees’ mental health: A systematic review. *PloS ONE.* 13. doi:10.1371/journal.pone.0191332

Kirby, J. N., and Gilbert, P. (2019). Commentary Regarding Wilson et al. (2018) “Effectiveness of ‘Self-Compassion’ Related Therapies: a Systematic Review and Meta-analysis.” All Is Not as It Seems. *Mindfulness.* 10, 1006-1016. doi:10.1007/s12671-018-1088-8

Laird, K. T., Paholpak, P., Roman, M., Rahi, B., and Lavretsky, H. (2018). Mind-Body Therapies for Late-Life Mental and Cognitive Health. ‎*Curr. Psychiatry Rep*. 20. doi:10.1007/s11920-018-0864-4

Levy, K. N., McMain, S., Bateman, A., and Clouthier, T. (2018). Treatment of Borderline Personality Disorder. *Psychiatr. Clin. North Am*. 41, 711-728. doi:10.1016/j.psc.2018.07.011

Li, S. Y. H., and Bressington, D. (2019). The effects of mindfulness-based stress reduction on depression, anxiety, and stress in older adults: A systematic review and meta-analysis. *Int. J. Ment. Health Nurs*. 28, 635-656. doi:10.1111/inm.12568

Liu, Z., Sun, Y. Y., and Zhong, B. L. (2018). Mindfulness-based stress reduction for family carers of people with dementia. *Cochrane Database Syst. Rev.* 2018. doi:10.1002/14651858.CD012791.pub2

Lo, K., Waterland, J., Todd, P., Gupta, T., Bearman, M., Hassed, C., et al. (2018). Group interventions to promote mental health in health professional education: a systematic review and meta-analysis of randomised controlled trials. *Adv. Health Sci. Educ*. 23, 413-447. doi:10.1007/s10459-017-9770-5

Lomas, T., Medina, J. C., Ivtzan, I., Rupprecht, S., and Eiroa-Orosa, F. J. (2018). Mindfulness-based interventions in the workplace: An inclusive systematic review and meta-analysis of their impact upon wellbeing. *J. Posit. Psychol*. doi:10.1080/17439760.2018.1519588

Lomas, T., Medina, J. C., Ivtzan, I., Rupprecht, S., and Eiroa-Orosa, F. J. (2019). A Systematic Review and Meta-analysis of the Impact of Mindfulness-Based Interventions on the Well-Being of Healthcare Professionals. *Mindfulness.* 10, 1193-1216. doi:10.1007/s12671-018-1062-5

López-Pinar, C., Martínez-Sanchís, S., Carbonell-Vayá, E., Sánchez-Meca, J., and Fenollar-Cortés, J. (2019). Efficacy of Nonpharmacological Treatments on Comorbid Internalizing Symptoms of Adults With Attention-Deficit/Hyperactivity Disorder: A Meta-Analytic Review. *J. Atten. Disord.* 13. doi:10.1177/1087054719855685

Loughnan, S. A., Wallace, M., Joubert, A. E., Haskelberg, H., Andrews, G., and Newby, J. M. (2018). A systematic review of psychological treatments for clinical anxiety during the perinatal period. *Arch. Women Ment. Hlth.* 21*,* 481-490. doi:10.1007/s00737-018-0812-7

Lovas, D. A., and Schuman-Olivier, Z. (2018). Mindfulness-based cognitive therapy for bipolar disorder: A systematic review. *J. Affect. Disord.* 240, 247-261. doi:10.1016/j.jad.2018.06.017

Mahoney, A., Karatzias, T., and Hutton, P. (2019). A systematic review and meta-analysis of group treatments for adults with symptoms associated with complex post-traumatic stress disorder. *J. Affect.Disord.*243, 305-321. doi:10.1016/j.jad.2018.09.059

McClintock, A. S., Rodriguez, M. A., and Zerubavel, N. (2019). The Effects of Mindfulness Retreats on the Psychological Health of Non-clinical Adults: a Meta-analysis. *Mindfulness*. 10, 1443-1454. doi:10.1007/s12671-019-01123-9

Mehta, M. (2018). New advances in cognitive behavioral therapy. *J. Indian Assoc. Child Adolesc. Ment. Health. 14*, 12-30.

Meister, K., and Juckel, G. (2018). A Systematic Review of Mechanisms of Change in Body-Oriented Yoga in Major Depressive Disorders. *Pharmacopsychiatry. 51*, 73-81. doi:10.1055/s-0043-111013

Mendelson, T., and Eaton, W. W. (2018). Recent advances in the prevention of mental disorders. *Soc Psychiatry Psychiatr. Epidemiol. 53*, 325-339. doi:10.1007/s00127-018-1501-6

Michaelis, R., Tang, V., Wagner, J. L., Modi, A. C., Curt LaFrance, W., Jr., Goldstein, L. H., et al. (2018). Cochrane systematic review and meta-analysis of the impact of psychological treatments for people with epilepsy on health-related quality of life. *Epilepsia. 59*, 315-332. doi:10.1111/epi.13989

Modica, C., and Hoenig, K. (2018). Mindfulness in Follow-Up Care after Breast Cancer: Can It Prevent Recurrence? *Breast Car.* 13, 102-108. doi:10.1159/000488716

Niknejad, B., Bolier, R., Henderson, C. R., Jr., Delgado, D., Kozlov, E., Löckenhoff, C. E., et al. (2018). Association between psychological interventions and chronic pain outcomes in older adults: A systematic review and meta-analysis. *JAMA Intern. Med.* 178, 830-839. doi:10.1001/jamainternmed.2018.0756

O'Connor, M., Munnelly, A., Whelan, R., and McHugh, L. (2018). The Efficacy and Acceptability of Third-Wave Behavioral and Cognitive eHealth Treatments: A Systematic Review and Meta-Analysis of Randomized Controlled Trials. *Behav. Ther*. 49, 459-475. doi:10.1016/j.beth.2017.07.007

Perach, R., Allen, C. K., Kapantai, I., Madrid-Valero, J. J., Miles, E., Charlton, R. A., et al. (2019). The psychological wellbeing outcomes of nonpharmacological interventions for older persons with insomnia symptoms: A systematic review and meta-analysis. *Sleep Med. Rev.* 43, 1-13. doi:10.1016/j.smrv.2018.09.003

Perich, T., and Mitchell, P. B. (2019). Psychological interventions for young people at risk for bipolar disorder: A systematic review. *J. Affect Disord.* 252, 84-91. doi:10.1016/j.jad.2019.04.058

Petrie, K., Crawford, J., Baker, S. T. E., Dean, K., Robinson, J., Veness, B. G., et al. (2019). Interventions to reduce symptoms of common mental disorders and suicidal ideation in physicians: a systematic review and meta-analysis. *Lancet Psychiat.* 6, 225-234. doi:10.1016/S2215-0366(18)30509-1

Prothero, L., Barley, E., Galloway, J., Georgopoulou, S., and Sturt, J. (2018). The evidence base for psychological interventions for rheumatoid arthritis: A systematic review of reviews. *Int. J. Nurs. Stud*. 82, 20-29. doi:10.1016/j.ijnurstu.2018.03.008

Robins, J. L. W. (2019). Should we meditate while we wait? *J. Womens Health.* 28, 109-110. doi:10.1089/jwh.2018.7576

Ruffault, A., Czernichow, S., Lurbe I Puerto, K., Fournier, J. F., Carette, C., et al. (2019). Mindfulness-based intervention among patients with obesity and binge eating disorder: Preliminary results of the MindOb randomized controlled trial. *Journal de Therapie Comportementale et Cognitive.* 29, 4-24. doi:10.1016/j.jtcc.2018.09.001

Saeed, S. A., Cunningham, K., and Bloch, R. M. (2019). Depression and anxiety disorders: Benefits of exercise, yoga, and meditation. *Am. Fam. Physician*. 99, 620-627.

Schell, L. K., Monsef, I., Wöckel, A., and Skoetz, N. (2019). Mindfulness-based stress reduction for women diagnosed with breast cancer. *Cochrane Database Syst. Rev.* 2019. doi:10.1002/14651858.CD011518.pub2

Schumer, M. C., Lindsay, E. K., and David Creswell, J. (2018). Brief mindfulness training for negative affectivity: A systematic review and meta-analysis. *J. Consult. Clin. Psychol*. 86, 569-583. doi:10.1037/ccp0000324

Scott-Sheldon, L. A. J., Balletto, B. L., Donahue, M. L., Feulner, M. M., Cruess, D. G., Salmoirago-Blotcher, E., et al. (2019). Mindfulness-Based Interventions for Adults Living with HIV/AIDS: A Systematic Review and Meta-analysis. *AIDS Behav.* 23, 60-75. doi:10.1007/s10461-018-2236-9

Selchen, S., Hawley, L. L., Regev, R., Richter, P., and Rector, N. A. (2018). Mindfulness-Based Cognitive Therapy for OCD: Stand-Alone and Post-CBT Augmentation Approaches. *Int. J. Cogn. Ther*. 11, 58-79. doi:10.1007/s41811-018-0003-3

Sesel, A. L., Sharpe, L., Beadnall, H. N., Barnett, M. H., Szabo, M., and Naismith, S. L. (2019). The evaluation of an online mindfulness program for people with multiple sclerosis: Study protocol. *BMC Neurology,* 19. doi:10.1186/s12883-019-1356-9

Sesel, A. L., Sharpe, L., and Naismith, S. L. (2018). Efficacy of Psychosocial Interventions for People with Multiple Sclerosis: A Meta-Analysis of Specific Treatment Effects. *Psychother. Psychosom.* 87, 105-111. doi:10.1159/000486806

Sevilla-Llewellyn-Jones, J., Santesteban-Echarri, O., Pryor, I., McGorry, P., and Alvarez-Jimenez, M. (2018). Web-based mindfulness interventions for mental health treatment: Systematic review and meta-analysis. *J. Med. Internet. Res.* 20. doi:10.2196/10278

Simpson, R., Simpson, S., Ramparsad, N., Lawrence, M., Booth, J., and Mercer, S. W. (2019). Mindfulness-based interventions for mental well-being among people with multiple sclerosis: A systematic review and meta-analysis of randomised controlled trials. *J. Neurol. Neurosurg. Psychiatry.* 13.doi:10.1136/jnnp-2018-320165

Singh, S. K., and Gorey, K. M. (2018). Relative effectiveness of mindfulness and cognitive behavioral interventions for anxiety disorders: Meta-analytic review. *Social Work Mental Health.* 16, 238-251. doi:10.1080/15332985.2017.1373266

Slimani, M., Bragazzi, N. L., Znazen, H., Paravlic, A., Azaiez, F., and Tod, D. (2018). Psychosocial predictors and psychological prevention of soccer injuries: A systematic review and meta-analysis of the literature. *Phys. Ther. Sport*. 32, 293-300. doi:10.1016/j.ptsp.2018.05.006

Smith, C. A., Shewamene, Z., Galbally, M., Schmied, V., and Dahlen, H. (2019). The effect of complementary medicines and therapies on maternal anxiety and depression in pregnancy: A systematic review and meta-analysis. *J. Affect. Disord.* 245, 428-439. doi:10.1016/j.jad.2018.11.054

Spinelli, C., Wisener, M., and Khoury, B. (2019). Mindfulness training for healthcare professionals and trainees: A meta-analysis of randomized controlled trials. *J. Psychosom. Res*. 120, 29-38. doi:10.1016/j.jpsychores.2019.03.003

Temple, J., Salmon, P., Tudur-Smith, C., Huntley, C. D., and Fisher, P. L. (2018). A systematic review of the quality of randomized controlled trials of psychological treatments for emotional distress in breast cancer. *J. Psychosom.c Res.*108, 22-31. doi:10.1016/j.jpsychores.2018.02.013

Tian, L., Zhang, Y., Li, L., Wu, Y., and Li, Y. (2019). The efficacy of mindfulness-based interventions for patients with COPD: A systematic review and meta-analysis protocol. *BMJ Open.* 9. doi:10.1136/bmjopen-2018-026061

Vakonaki, E., Tsiminikaki, K., Plaitis, S., Fragkiadaki, P., Tsoukalas, D., Katsikantami, I., et al. (2018). Common mental disorders and association with telomere length (Review). *Biomed. Rep.* 8, 111-116. doi:10.3892/br.2018.1040

Vasudev, A., Torres-Platas, S. G., Kerfoot, K., Potes, A., Therriault, J., Gifuni, A.,et al. (2019). Mind-Body Interventions in Late-Life Mental Illnesses and Cognitive Disorders: A Narrative Review. *Am. J. Geriatr. Psychiatry.* 27, 536-547. doi:10.1016/j.jagp.2018.10.020

Wilson, A. C., Mackintosh, K., Power, K., and Chan, S. W. Y. (2019). Effectiveness of Self-Compassion Related Therapies: a Systematic Review and Meta-analysis. *Mindfulness.* 10, 979-995. doi:10.1007/s12671-018-1037-6

Witt, K., Boland, A., Lamblin, M., McGorry, P. D., Veness, B., Cipriani, A., et al. (2019). Effectiveness of universal programmes for the prevention of suicidal ideation, behaviour and mental ill health in medical students: A systematic review and meta-analysis. *Evid. Based Ment. Health*. 22, 84-90. doi:10.1136/ebmental-2019-300082

Yusufov, M., Nicoloro-SantaBarbara, J., Grey, N. E., Moyer, A., and Lobel, M. (2019). Meta-analytic evaluation of stress reduction interventions for undergraduate and graduate students. *Int. J. Stress. Manag*. 26, 132-145. doi:10.1037/str0000099

Zhang, Q., Zhao, H., and Zheng, Y. (2019). Effectiveness of mindfulness-based stress reduction (MBSR) on symptom variables and health-related quality of life in breast cancer patients—a systematic review and meta-analysis. *Support. Care Cancer.* 27, 771-781. doi:10.1007/s00520-018-4570-x

Zill, J. M., Christalle, E., Tillenburg, N., Mrowietz, U., Augustin, M., Härter, M., et al. (2018). Effects of psychosocial interventions on patient-reported outcomes in patients with psoriasis: a systematic review and meta-analysis. *B. J. Dermatol.* doi:10.1111/bjd.17272

Zou, L., Yeung, A., Quan, X., Hui, S. S. C., Hu, X., Chan, J. S. M., et al. (2018). Mindfulness-based baduanjin exercise for depression and anxiety in people with physical or mental illnesses: A systematic review and meta-analysis. *Int. J. Env. Res. Pub. He. 15*. doi:10.3390/ijerph15020321

**2 Supplementary material 2: Excluded studies on Cognitive behavioral therapy with virtual reality-based exposure (CBT-VRexp)**

| **Study** | **Main reason for exclusion** |
| --- | --- |
| Banneyer et al., 2018 | Limited population scope |
| Chesham et al., 2018 | Limited in clinical scope |
| Fernández-Álvarez et al., 2019 | Limited to deterioration rates |
| Fodor et al., 2018 | Not specific to CBT with VR exposure |
| Gujjar et al., 2019 | Not a meta-analysis |
| Lindner et al., 2019 | Not a meta-analysis |
| Ma et al., 2019 | Not about CBT-VRexp |
| Riva et al., 2019 | Not a meta-analysis |
| Scheffler et al., 2018 | Not for anxiety disorders |
| Thabrew et al., 2018 | Not about CBT-VRexp |

**References**

Banneyer, K. N., Bonin, L., Price, K., Goodman, W. K., and Storch, E. A. (2018). Cognitive Behavioral Therapy for Childhood Anxiety Disorders: a Review of Recent Advances. *Curr. Psychiatry Rep*. 20. doi:10.1007/s11920-018-0924-9

Chesham, R. K., Malouff, J. M., and Schutte, N. S. (2018). Meta-Analysis of the Efficacy of Virtual Reality Exposure Therapy for Social Anxiety. *Behav. Change*. 35, 152-166. doi:10.1017/bec.2018.15

Fernández-Álvarez, J., Rozental, A., Carlbring, P., Colombo, D., Riva, G., Anderson, P. L., et al., (2019). Deterioration rates in Virtual Reality Therapy: An individual patient data level meta-analysis. *J. Anxiety Disord*. 61, 3-17. doi:10.1016/j.janxdis.2018.06.005

Fodor, L. A., Coteţ, C. D., Cuijpers, P., Szamoskozi, S., David, D., and Cristea, I. A. (2018). The effectiveness of virtual reality based interventions for symptoms of anxiety and depression: A meta-Analysis. *Sci. Rep.* 8. doi:10.1038/s41598-018-28113-6

Gujjar, K. R., van Wijk, A., Kumar, R., and de Jongh, A. (2019). Are Technology-Based Interventions Effective in Reducing Dental Anxiety in Children and Adults? A Systematic Review. *J. Evid. Based Dent. Pract.* 19, 140-155. doi:10.1016/j.jebdp.2019.01.009

Lindner, P., Miloff, A., Fagernäs, S., Andersen, J., Sigeman, M., Andersson, G., et al. (2019). Corrigendum to “Therapist-led and self-led one-session virtual reality exposure therapy for public speaking anxiety with consumer hardware and software: A randomized controlled trial” [J. Anxiety Disord. 61 (2) (2019) 45–54] (Journal of Anxiety Disorders (2019) 61 (45–54), (S0887618517306321), (10.1016/j.janxdis.2018.07.003)). *J. Anxiety Dis.* 64, 90. doi:10.1016/j.janxdis.2019.04.002

Ma, T., Chattopadhyay, D., and Sharifi, H. (2019). Virtual humans in health-related interventions: A meta-analysis. In: Proceeding CHI EA '19 Extended Abstracts of the 2019 CHI Conference on Human Factors in Computing Systems Paper No. LBW1717; 2019 May 4-9; Glasgow, Scotland Uk; New York: ACM (2019). doi:10.1145/3290607.3312853

Riva, G., Wiederhold, B. K., and Mantovani, F. (2019). Neuroscience of Virtual Reality: From Virtual Exposure to Embodied Medicine. *Cyberpsychol. Behav. Soc. Netw*. 22, 82-96. doi:10.1089/cyber.2017.29099.gri

Scheffler, M., Koranyi, S., Meissner, W., Strauß, B., and Rosendahl, J. (2018). Efficacy of non-pharmacological interventions for procedural pain relief in adults undergoing burn wound care: A systematic review and meta-analysis of randomized controlled trials. *Burns.* 44, 1709-1720. doi:10.1016/j.burns.2017.11.019

Thabrew, H., Stasiak, K., Hetrick, S. E., Wong, S., Huss, J. H., and Merry, S. N. (2018). E-Health interventions for anxiety and depression in children and adolescents with long-term physical conditions. *Cochrane Database Syst. Rev*. 2018. doi:10.1002/14651858.CD012489.pub2

**3 Supplementary material 3: Excluded studies on Cognitive-behavioral therapy (CBT)**

| **Study** | **Main reason for exclusion** |
| --- | --- |
| Andersson et al., 2019 | Limited intervention modality |
| Andrews et al., 2018 | Limited intervention modality |
| Aylett et al., 2018 | Not about CBT |
| Banneyer et al., 2018 | Limited population scope |
| Barry et al., 2018 | No information about RCT |
| Benbow and Anderson, 2019 | About CBT-VRexp |
| Bernard et al., 2018 | CBT + added intervention |
| Bernardy et al., 2018 | Not for anxiety disorders |
| Birnie et al., 2018 | Limited population scope |
| Boschloo et al., 2019 | Not for anxiety disorders |
| Brand et al., 2018 | Not for anxiety disorders |
| Carl et al., 2019 | About CBT-VRexp |
| Carlbring et al., 2018 | Limited intervention modality |
| Carnes et al., 2019 | Limited population scope |
| Cavanagh etal., 2018 | Not a meta-analysis |
| Chan et al., 2018 | Not a meta-analysis |
| Chesham et al., 2018 | About CBT-VRexp |
| de Abreu Costa et al., 2019 | About MBI |
| Elliott and Place, 2019 | Not a meta-analysis |
| Fernández-Álvarez et al., 2019 | About CBT-VRexp |
| Fordham et al., 2018 | Not a meta-analysis |
| Ghielen et al., 2019 | Limited population scope |
| Giummarra et al., 2018 | Limited population scope |
| Gottschalk et al., 2019 | Not a meta-analysis |
| Gregory Jr, 2019 | Limited population scope |
| Grist et al., 2019 | Limited population scope |
| Gujjar et al., 2019 | Not for anxiety disorders |
| Güven and Gökçe, 2018 | Not about treatment efficacy |
| Hanlon et al., 2018 | Limited population scope |
| Harada et al., 2018 | Not for anxiety disorders |
| Heyman et al., 2018 | Not for anxiety disorders |
| J. Huang et al., 2018 | Limited population scope |
| L. Huang et al., 2018 | Limited population scope |
| James et al., 2018 | Limited population scope |
| Jiang et al., 2018 | Limited population scope |
| Jones et al., 2018 | Not for anxiety disorders |
| Kaddour et al., 2018 | Limited population scope |
| Kayrouz et al., 2018 | Limited population scope |
| Kazantzis et al., 2018 | Not about treatment efficacy |
| Kim and Kim, 2018 | Not about CBT |
| Kishita et al., 2018 | Limited population scope |
| Kolubinski et al., 2018 | Not for anxiety disorders |
| Kreuze et al., 2018 | Limited population scope |
| Law et al., 2019 | Limited population scope |
| Lewey et al., 2018 | Limited population scope |
| Linardon et al., 2018 | Not about CBT |
| Lindner et al., 2019 | Not a meta-analysis |
| Liu et al., 2019 | Not for anxiety disorders |
| Lopez et al., 2018 | Not for anxiety disorders |
| López-Pinar et al., 2019 | Not for anxiety disorders |
| Maguire et al., 2018 | Limited population scope |
| Marker and Norton, 2018 | CBT + added intervention |
| Maynard et al., 2018 | Not for anxiety disorders |
| Mehta et al., 2019 | Not for anxiety disorders |
| Meng et al., 2019 | Not a meta-analysis |
| Menzies et al., 2018 | Not for anxiety disorders |
| Morris et al., 2018 | Not about CBT |
| Nair et al., 2018 | Not for anxiety disorders |
| Natale et al., 2019 | Not for anxiety disorders |
| Noble and Marshman, 2018 | Limited population scope |
| O'Connor et al., 2018 | Limited intervention modality |
| Perihan et al., 2019 | Limited population scope |
| Purgato et al., 2018 | Limited population scope |
| Radu, Moldovan, Pintea, Băban, & Dumitrașcu, 2018 | Not for anxiety disorders |
| Reavell et al., 2018 | Limited population scope |
| Rith-Najarian et al., 2019 | Limited population scope |
| Riva et al., 2019 | About CBT-VRexp |
| Roquet and Monfils, 2018 | Not about CBT |
| Rozental et al., 2019 | Limited intervention modality |
| Rozental et al., 2018 | Not for anxiety disorders |
| Salomonsson et al., 2018 | Not for anxiety disorders |
| Savoia and Sztamfater, 2018 | Not a meta-analysis |
| Schäfer et al., 2018 | Not a meta-analysis |
| Schwartze et al., 2019 | Limited in clinical scope |
| Selchen et al., 2018 | Not a meta-analysis |
| Sereda et al., 2018 | Not about CBT |
| Sesel et al., 2018 | Not for anxiety disorders |
| Shivakumar et al., 2019 | Not for anxiety disorders |
| Skjernov et al., 2018 | Not a meta-analysis |
| Springer et al., 2018 | Limited in clinical scope |
| Stain et al., 2019 | Not for anxiety disorders |
| Stevens et al., 2019 | Not for anxiety disorders |
| Sun et al., 2019 | Limited population scope |
| Tang et al., 2018 | Limited population scope |
| Thabrew et al., 2018 | Limited population scope |
| Thabrew et al., 2018 | Limited population scope |
| Walczak et al., 2018 | Limited population scope |
| L. Wang et al., 2018 | Not about CBT |
| S. B. Wang et al., 2018 | Not for anxiety disorders |
| Werner-Seidler et al., 2018 | Not for anxiety disorders |
| Ye et al., 2018 | Limited population scope |
| Zaboski and Storch, 2018 | Not about CBT |
| Zhang, Borhneimer, et al., 2019 | Limited intervention modality, no information on specific anxiety disorders |
| Zhang, Franklin, et al., 2019 | Limited intervention modality, no information on specific anxiety disorders |
| Zhou et al., 2019 | Limited population scope |

**References**

Andersson, G., Carlbring, P., Titov, N., and Lindefors, N. (2019). Internet Interventions for Adults with Anxiety and Mood Disorders: A Narrative Umbrella Review of Recent Meta-Analyses. *Can. J. Psychiatry.* 64, 465-470. doi:10.1177/0706743719839381

Andrews, G., Basu, A., Cuijpers, P., Craske, M. G., McEvoy, P., English, C. L., et al., (2018). Computer therapy for the anxiety and depression disorders is effective, acceptable and practical health care: An updated meta-analysis. *J. Anxiety Dis*. 55, 70-78. doi:10.1016/j.janxdis.2018.01.001

Aylett, E., Small, N., and Bower, P. (2018). Exercise in the treatment of clinical anxiety in general practice - A systematic review and meta-analysis. *BMC Health Serv. Res*. 18. doi:10.1186/s12913-018-3313-5

Banneyer, K. N., Bonin, L., Price, K., Goodman, W. K., and Storch, E. A. (2018). Cognitive Behavioral Therapy for Childhood Anxiety Disorders: a Review of Recent Advances. *Curr. Psychiatry Rep.* 20. doi:10.1007/s11920-018-0924-9

Barry, T. J., Yeung, S. P., and Lau, J. Y. F. (2018). Meta-analysis of the influence of age on symptom change following cognitive-behavioural treatment for anxiety disorders. *J. Adolesc*. 68, 232-241. doi:10.1016/j.adolescence.2018.08.008

Benbow, A. A., and Anderson, P. L. (2019). A meta-analytic examination of attrition in virtual reality exposure therapy for anxiety disorders. *J. Anxiety Dis.* 61, 18-26. doi:10.1016/j.janxdis.2018.06.006

Bernard, P., Romain, A. J., Caudroit, J., Chevance, G., Carayol, M., Gourlan, M., et al., (2018). Cognitive behavior therapy combined with exercise for adults with chronic diseases: Systematic review and meta-analysis. *Health Psychol*. 37, 433-450. doi:10.1037/hea0000578

Bernardy, K., Klose, P., Welsch, P., and Häuser, W. (2018). Efficacy, acceptability and safety of cognitive behavioural therapies in fibromyalgia syndrome – A systematic review and meta-analysis of randomized controlled trials. *Eur. J. Pain*, 22, 242-260. doi:10.1002/ejp.1121

Birnie, K. A., Noel, M., Chambers, C. T., Uman, L. S., and Parker, J. A. (2018). Psychological interventions for needle-related procedural pain and distress in children and adolescents. *Cochrane Database Syst. Rev*. 2018. doi:10.1002/14651858.CD005179.pub4

Boschloo, L., Bekhuis, E., Weitz, E. S., Reijnders, M., DeRubeis, R. J., Dimidjian, S.,et al., (2019). The symptom-specific efficacy of antidepressant medication vs. cognitive behavioral therapy in the treatment of depression: results from an individual patient data meta-analysis. *World Psychiatry.* 18, 183-191. doi:10.1002/wps.20630

Brand, R. M., McEnery, C., Rossell, S., Bendall, S., and Thomas, N. (2018). Do trauma-focussed psychological interventions have an effect on psychotic symptoms? A systematic review and meta-analysis. *Schizophr. Res*. 195, 13-22. doi:10.1016/j.schres.2017.08.037

Carl, E., Stein, A. T., Levihn-Coon, A., Pogue, J. R., Rothbaum, B., Emmelkamp, P., et al., (2019). Virtual reality exposure therapy for anxiety and related disorders: A meta-analysis of randomized controlled trials. *J. Anxiety Dis*., 61, 27-36. doi:10.1016/j.janxdis.2018.08.003

Carlbring, P., Andersson, G., Cuijpers, P., Riper, H., and Hedman-Lagerlöf, E. (2018). Internet-based vs. face-to-face cognitive behavior therapy for psychiatric and somatic disorders: an updated systematic review and meta-analysis. *Cogn. Behav. Ther*. 47, 1-18. doi:10.1080/16506073.2017.1401115

Carnes, A., Matthewson, M., and Boer, O. (2019). The contribution of parents in childhood anxiety treatment: A meta-analytic review. *Clin. Psychol*. doi:10.1111/cp.12179

Cavanagh, K., Herbeck Belnap, B., Rothenberger, S. D., Abebe, K. Z., and Rollman, B. L. (2018). My care manager, my computer therapy and me: The relationship triangle in computerized cognitive behavioural therapy. *Internet Inter*. 11, 11-19. doi:10.1016/j.invent.2017.10.005

Chan, P., Bhar, S., Davison, T. E., Doyle, C., Knight, B. G., Koder, D., et al., (2018). Characteristics of cognitive behavioral therapy for older adults living in residential care: Protocol for a systematic review. *J. Med. Internet Res*. 20. doi:10.2196/resprot.9902

Chesham, R. K., Malouff, J. M., and Schutte, N. S. (2018). Meta-Analysis of the Efficacy of Virtual Reality Exposure Therapy for Social Anxiety. *Behav. Change.* 35, 152-166. doi:10.1017/bec.2018.15

de Abreu Costa, M., D’Alò de Oliveira, G. S., Tatton-Ramos, T., Manfro, G. G., and Salum, G. A. (2019). Anxiety and Stress-Related Disorders and Mindfulness-Based Interventions: a Systematic Review and Multilevel Meta-analysis and Meta-Regression of Multiple Outcomes. *Mindfulness.* 10, 996-1005. doi:10.1007/s12671-018-1058-1

Elliott, J. G., and Place, M. (2019). Practitioner Review: School refusal: developments in conceptualisation and treatment since 2000. *J. Child Psychol. Psychiatry*. 60, 4-15. doi:10.1111/jcpp.12848

Fernández-Álvarez, J., Rozental, A., Carlbring, P., Colombo, D., Riva, G., Anderson, P. L., et al., (2019). Deterioration rates in Virtual Reality Therapy: An individual patient data level meta-analysis. *J. Anxiety Dis*. 61, 3-17. doi:10.1016/j.janxdis.2018.06.005

Fordham, B., Sugavanam, T., Hopewell, S., Hemming, K., Howick, J., Kirtley, S., et al. (2018). Effectiveness of cognitive-behavioural therapy: A protocol for an overview of systematic reviews and meta-analyses. *BMJ Open.* 8. doi:10.1136/bmjopen-2018-025761

Ghielen, I., Rutten, S., Boeschoten, R. E., Houniet-de Gier, M., van Wegen, E. E. H., van den Heuvel, O. A., et al. (2019). The effects of cognitive behavioral and mindfulness-based therapies on psychological distress in patients with multiple sclerosis, Parkinson's disease and Huntington's disease: Two meta-analyses. *J. Psychosom. Res*. 122, 43-51. doi:10.1016/j.jpsychores.2019.05.001

Giummarra, M. J., Lennox, A., Dali, G., Costa, B., and Gabbe, B. J. (2018). Early psychological interventions for posttraumatic stress, depression and anxiety after traumatic injury: A systematic review and meta-analysis*. Clin. Psychol. Rev*. 62, 11-36. doi:10.1016/j.cpr.2018.05.001

Gottschalk, M. G., Richter, J., Ziegler, C., Schiele, M. A., Mann, J., Geiger, M. J., et al. (2019). Orexin in the anxiety spectrum: association of a HCRTR1 polymorphism with panic disorder/agoraphobia, CBT treatment response and fear-related intermediate phenotypes. *Transl. Psychiat*. 9. doi:10.1038/s41398-019-0415-8

Gregory Jr, V. L. (2019). Cognitive-Behavioral Therapy for Anxious Symptoms in Persons of African Descent: A Meta-Analysis. *J. Soc. Service Res.* 45, 87-101. doi:10.1080/01488376.2018.1479344

Grist, R., Croker, A., Denne, M., and Stallard, P. (2019). Technology Delivered Interventions for Depression and Anxiety in Children and Adolescents: A Systematic Review and Meta-analysis. *Clin. Child Fam. Psychol. Rev*. 22, 147-171. doi:10.1007/s10567-018-0271-8

Gujjar, K. R., van Wijk, A., Kumar, R., and de Jongh, A. (2019). Are Technology-Based Interventions Effective in Reducing Dental Anxiety in Children and Adults? A Systematic Review. *J. Evid. Based Dent. Prac.* 19, 140-155. doi:10.1016/j.jebdp.2019.01.009

Güven, E., and Gökçe, G. (2018). How effective are efficiency studies?: An evaluation in the context of cognitive behavioral therapies. *Turk Psikiyatri Dergisi*. 29. doi:10.5080/u22706

Hanlon, I., Hewitt, C., Bell, K., Phillips, A., and Mikocka-Walus, A. (2018). Systematic review with meta-analysis: online psychological interventions for mental and physical health outcomes in gastrointestinal disorders including irritable bowel syndrome and inflammatory bowel disease. *Aliment. Pharmacol. Ther*. 48, 244-259. doi:10.1111/apt.14840

Harada, T., Tsutomi, H., Mori, R., and Wilson, D. B. (2018). Cognitive-behavioural treatment for amphetamine-type stimulants (ATS)-use disorders. *Cochrane Database Syst. Rev*. 2018. doi:10.1002/14651858.CD011315.pub2

Heyman, R. E., Wojda, A. K., Eddy, J. M., Haydt, N. C., Geiger, J. F., and Slep, A. M. S. (2018). Dentist-Perceived Barriers and Attractors to Cognitive-Behavioral Treatment Provided by Mental Health Providers in Dental Practices. *Adv. Den. Res*. 29, 35-41. doi:10.1177/0022034517737023

Huang, J., Nigatu, Y. T., Smail-Crevier, R., Zhang, X., and Wang, J. (2018). Interventions for common mental health problems among university and college students: A systematic review and meta-analysis of randomized controlled trials. *J. Psychiatr. Res.* 107, 1-10. doi:10.1016/j.jpsychires.2018.09.018

Huang, L., Zhao, Y., Qiang, C., and Fan, B. (2018). Is cognitive behavioral therapy a better choice for women with postnatal depression? A systematic review and meta-analysis. *PloS ONE*. 13. doi:10.1371/journal.pone.0205243

James, A. C., Reardon, T., Soler, A., James, G., and Creswell, C. (2018). Cognitive behavioural therapy for anxiety disorders in children and adolescents. *Cochrane Database Syst. Rev.* 2018. doi:10.1002/14651858.CD013162

Jiang, Y., Shorey, S., Seah, B., Chan, W. X., Tam, W. W. S., and Wang, W. (2018). The effectiveness of psychological interventions on self-care, psychological and health outcomes in patients with chronic heart failure—A systematic review and meta-analysis. *Int. J. Nurs. Stud.* 78, 16-25. doi:10.1016/j.ijnurstu.2017.08.006

Jones, C., Hacker, D., Meaden, A., Cormac, I., Irving, C. B., Xia, J., et al. Chen, J. (2018). Cognitive behavioural therapy plus standard care versus standard care plus other psychosocial treatments for people with schizophrenia. *Cochrane Database Syst. Rev.* 2018. doi:10.1002/14651858.CD008712.pub3

Kaddour, L., Kishita, N., and Schaller, A. (2018). A meta-analysis of low-intensity cognitive behavioral therapy-based interventions for dementia caregivers. *Int. Psychogeriatr*. 12,1-16. doi:10.1017/S1041610218001436

Kayrouz, R., Dear, B. F., Kayrouz, B., Karin, E., Gandy, M., and Titov, N. (2018). Meta-analysis of the efficacy and acceptability of cognitive-behavioural therapy for Arab adult populations experiencing anxiety, depression or post-traumatic stress disorder. *Cogn. Behav. Ther*. 47, 412-430. doi:10.1080/16506073.2018.1445124

Kazantzis, N., Luong, H. K., Usatoff, A. S., Impala, T., Yew, R. Y., and Hofmann, S. G. (2018). The processes of cognitive behavioral therapy: A review of meta-analyses. *Cogn. Ther. Res.* 42, 349-357. doi:10.1007/s10608-018-9920-y

Kim, H. S., and Kim, E. J. (2018). Effects of Relaxation Therapy on Anxiety Disorders: A Systematic Review and Meta-analysis. *Arch. Psychiatr. Nurs.* 32, 278-284. doi:10.1016/j.apnu.2017.11.015

Kishita, N., Hammond, L., Dietrich, C. M., and Mioshi, E. (2018). Which interventions work for dementia family carers?: An updated systematic review of randomized controlled trials of carer interventions. *Int. Psychogeriatr*. 30, 1679-1696. doi:10.1017/S1041610218000947

Kolubinski, D. C., Frings, D., Nikčević, A. V., Lawrence, J. A., and Spada, M. M. (2018). A systematic review and meta-analysis of CBT interventions based on the Fennell model of low self-esteem. *Psychiatry Res.* 267, 296-305. doi:10.1016/j.psychres.2018.06.025

Kreuze, L. J., Pijnenborg, G. H. M., de Jonge, Y. B., and Nauta, M. H. (2018). Cognitive-behavior therapy for children and adolescents with anxiety disorders: A meta-analysis of secondary outcomes. *J. Anxiety Dis.* 60, 43-57. doi:10.1016/j.janxdis.2018.10.005

Law, E., Fisher, E., Eccleston, C., and Palermo, T. M. (2019). Psychological interventions for parents of children and adolescents with chronic illness. *Cochrane Database Syst. Rev*. 2019. doi:10.1002/14651858.CD009660.pub4

Lewey, J. H., Smith, C. L., Burcham, B., Saunders, N. L., Elfallal, D., and O’Toole, S. K. (2018). Comparing the Effectiveness of EMDR and TF-CBT for Children and Adolescents: a Meta-Analysis. *J. Child Adolesc. Trauma*. 11, 457-472. doi:10.1007/s40653-018-0212-1

Linardon, J., Fitzsimmons-Craft, E. E., Brennan, L., Barillaro, M., and Wilfley, D. E. (2018). Dropout from interpersonal psychotherapy for mental health disorders: A systematic review and meta-analysis. *Psychother. Res*. 1-12. doi:10.1080/10503307.2018.1497215

Lindner, P., Miloff, A., Fagernäs, S., Andersen, J., Sigeman, M., Andersson, G., et al. (2019). Corrigendum to “Therapist-led and self-led one-session virtual reality exposure therapy for public speaking anxiety with consumer hardware and software: A randomized controlled trial” [J. Anxiety Disord. 61 (2) (2019) 45–54] (Journal of Anxiety Disorders (2019) 61 (45–54), (S0887618517306321), (10.1016/j.janxdis.2018.07.003)). *J. Anxiety Dis.* 64, 90. doi:10.1016/j.janxdis.2019.04.002

Liu, J., Gill, N. S., Teodorczuk, A., Li, Z. J., and Sun, J. (2019). The efficacy of cognitive behavioural therapy in somatoform disorders and medically unexplained physical symptoms: A meta-analysis of randomized controlled trials. *J. Affect. Disord.* 245, 98-112. doi:10.1016/j.jad.2018.10.114

López-Pinar, C., Martínez-Sanchís, S., Carbonell-Vayá, E., Sánchez-Meca, J., and Fenollar-Cortés, J. (2019). Efficacy of Nonpharmacological Treatments on Comorbid Internalizing Symptoms of Adults With Attention-Deficit/Hyperactivity Disorder: A Meta-Analytic Review. *J. Atten. Disord.* doi:10.1177/1087054719855685

Lopez, P. L., Torrente, F. M., Ciapponi, A., Lischinsky, A. G., Cetkovich-Bakmas, M., Rojas, J. I., et al. (2018). Cognitive-behavioural interventions for attention deficit hyperactivity disorder (ADHD) in adults. *Cochrane Database Syst Rev.* 2018. doi:10.1002/14651858.CD010840.pub2

Maguire, P. N., Clark, G. I., and Wootton, B. M. (2018). The efficacy of cognitive behavior therapy for the treatment of perinatal anxiety symptoms: A preliminary meta-analysis. *J. Anxiety Disord*. 60, 26-34. doi:10.1016/j.janxdis.2018.10.002

Marker, I., & Norton, P. J. (2018). The efficacy of incorporating motivational interviewing to cognitive behavior therapy for anxiety disorders: A review and meta-analysis. *Clin. Psychol. Rev.* 62, 1-10. doi:10.1016/j.cpr.2018.04.004

Maynard, B. R., Heyne, D., Brendel, K. E., Bulanda, J. J., Thompson, A. M., and Pigott, T. D. (2018). Treatment for School Refusal Among Children and Adolescents: A Systematic Review and Meta-Analysis. *Res. Soc. Work Pract*. 28, 56-67. doi:10.1177/1049731515598619

Mehta, S., Peynenburg, V. A., and Hadjistavropoulos, H. D. (2019). Internet-delivered cognitive behaviour therapy for chronic health conditions: a systematic review and meta-analysis. *J. Behav. Med.* 42, 169-187. doi:10.1007/s10865-018-9984-x

Meng, F. Q., Han, H. Y., Luo, J., Liu, J., Liu, Z. R., Tang, Y., et al. (2019). Efficacy of cognitive behavioural therapy with medication for patients with obsessive-compulsive disorder: A multicentre randomised controlled trial in China. *J. Affect Disord*, 253, 184-192. doi:10.1016/j.jad.2019.04.090

Menzies, R. E., Zuccala, M., Sharpe, L., and Dar-Nimrod, I. (2018). The effects of psychosocial interventions on death anxiety: A meta-analysis and systematic review of randomised controlled trials. *J Anxiety Disord*. 59, 64-73. doi:10.1016/j.janxdis.2018.09.004

Morris, L., Stander, J., Ebrahim, W., Eksteen, S., Meaden, O. A., Ras, A., et al. (2018). Effect of exercise versus cognitive behavioural therapy or no intervention on anxiety, depression, fitness and quality of life in adults with previous methamphetamine dependency: a systematic review. *Addict Sci. Clin. Pract*. 13, 4. doi:10.1186/s13722-018-0106-4

Nair, U., Armfield, N. R., Chatfield, M. D., and Edirippulige, S. (2018). The effectiveness of telemedicine interventions to address maternal depression: A systematic review and meta-analysis. *J.Telemed. Telecare*. 24, 639-650. doi:10.1177/1357633X18794332

Natale, P., Ruospo, M., Saglimbene, V. M., Palmer, S. C., and Strippoli, G. F. M. (2019). Interventions for improving sleep quality in people with chronic kidney disease. *Cochrane Database Syst Rev*. 2019. doi:10.1002/14651858.CD012625.pub2

Noble, F., and Marshman, Z. (2018). The effectiveness of Cognitive Behavioural Therapy in the reduction of dental anxiety in children. *Evid. Based Dent*. 19, 104. doi:10.1038/sj.ebd.6401339

O'Connor, M., Munnelly, A., Whelan, R., and McHugh, L. (2018). The Efficacy and Acceptability of Third-Wave Behavioral and Cognitive eHealth Treatments: A Systematic Review and Meta-Analysis of Randomized Controlled Trials. *Behav Ther.* 49, 459-475. doi:10.1016/j.beth.2017.07.007

Perihan, C., Burke, M., Bowman-Perrott, L., Bicer, A., Gallup, J., Thompson, J., et al. (2019). Effects of Cognitive Behavioral Therapy for Reducing Anxiety in Children with High Functioning ASD: A Systematic Review and Meta-Analysis. *J. Autism Dev. Disord*.. doi:10.1007/s10803-019-03949-7

Purgato, M., Gastaldon, C., Papola, D., van Ommeren, M., Barbui, C., and Tol, W. A. (2018). Psychological therapies for the treatment of mental disorders in low- and middle-income countries affected by humanitarian crises. *Cochrane Database Syst. Rev*. 2018. doi:10.1002/14651858.CD011849.pub2

Radu, M., Moldovan, R., Pintea, S., Băban, A., and Dumitrașcu, D. (2018). Predictors of outcome in cognitive and behavioural interventions for irritable bowel syndrome. A meta-analysis. *J. Gastrointestin. Liver Dis.* 27, 257-263. doi:10.15403/jgld.2014.1121.273.bab

Reavell, J., Hopkinson, M., Clarkesmith, D., and Lane, D. A. (2018). Effectiveness of cognitive behavioral therapy for depression and anxiety in patients with cardiovascular disease: A systematic review and meta-analysis. *Psychosom. Med*. 80, 742-753. doi:10.1097/PSY.0000000000000626

Rith-Najarian, L. R., Mesri, B., Park, A. L., Sun, M., Chavira, D. A., and Chorpita, B. F. (2019). Durability of Cognitive Behavioral Therapy Effects for Youth and Adolescents With Anxiety, Depression, or Traumatic Stress:A Meta-Analysis on Long-Term Follow-Ups. *Behav. Ther*. 50, 225-240. doi:10.1016/j.beth.2018.05.006

Riva, G., Wiederhold, B. K., and Mantovani, F. (2019). Neuroscience of Virtual Reality: From Virtual Exposure to Embodied Medicine. *Cyberpsychol. Behav. Soc. Netw*. 22, 82-96. doi:10.1089/cyber.2017.29099.gri

Roquet, R. F., and Monfils, M. H. (2018). Does exercise augment operant and Pavlovian extinction: A meta-analysis. *J. Psychiatr. Res*. 96, 73-93. doi:10.1016/j.jpsychires.2017.09.018

Rozental, A., Andersson, G., and Carlbring, P. (2019). In the absence of effects: An individual patient data meta-analysis of non-response and its predictors in internet-based cognitive behavior therapy. *Front. Psychol*, 10. doi:10.3389/fpsyg.2019.00589

Rozental, A., Bennett, S., Forsström, D., Ebert, D. D., Shafran, R., Andersson, G., et al. (2018). Targeting procrastination using psychological treatments: A systematic review and meta-analysis. *Front. Psychol*. 9. doi:10.3389/fpsyg.2018.01588

Salomonsson, S., Hedman-Lagerlöf, E., and Öst, L. G. (2018). Sickness absence: A systematic review and meta-analysis of psychological treatments for individuals on sick leave due to common mental disorders. *Psychol. Med.* 48, 1954-1965. doi:10.1017/S0033291718000065

Savoia, M. G., and Sztamfater, S. (2018). Clinical implications of social anxiety disorder treatment with cognitive behavioral group therapy. In: Social Anxiety Disorder: Recognition, Diagnosis and Management; NICE Clinical guideline; Published: 22 May 2013; p.151-162.

Schäfer, S. K., Ihmig, F. R., Lara H, K. A., Neurohr, F., Kiefer, S., Staginnus, M. et al. (2018). Effects of heart rate variability biofeedback during exposure to fear-provoking stimuli within spider-fearful individuals: Study protocol for a randomized controlled trial. *Trials*. 19. doi:10.1186/s13063-018-2554-2

Schwartze, D., Barkowski, S., Strauss, B., Knaevelsrud, C., and Rosendahl, J. (2019). Efficacy of group psychotherapy for posttraumatic stress disorder: Systematic review and meta-analysis of randomized controlled trials. *Psychother. Res*. 29, 415-431. doi:10.1080/10503307.2017.1405168

Selchen, S., Hawley, L. L., Regev, R., Richter, P., and Rector, N. A. (2018). Mindfulness-Based Cognitive Therapy for OCD: Stand-Alone and Post-CBT Augmentation Approaches. *International J. Cogn. Ther.* 11, 58-79. doi:10.1007/s41811-018-0003-3

Sereda, M., Xia, J., El Refaie, A., Hall, D. A., and Hoare, D. J. (2018). Sound therapy (using amplification devices and/or sound generators) for tinnitus. *Cochrane Database Syst Rev.* 2018. doi:10.1002/14651858.CD013094.pub2

Sesel, A. L., Sharpe, L., and Naismith, S. L. (2018). Efficacy of Psychosocial Interventions for People with Multiple Sclerosis: A Meta-Analysis of Specific Treatment Effects. *Psychother Psychosom*. 87, 105-111. doi:10.1159/000486806

Shivakumar, V., Dinakaran, D., Narayanaswamy, J., and Venkatasubramanian, G. (2019). Noninvasive brain stimulation in obsessive-compulsive disorder. *Indian J Psychiatry*. 61, S66-S76. doi:10.4103/psychiatry.IndianJPsychiatry_522_18

Skjernov, M., Fink, P., Fallon, B., Rasmussen, F., and Simonsen, E. (2018). Feasibility study of group cognitive behavioral therapy for severe health anxiety. *J. Cogn. Psychother*. 32, 223-240. doi:10.1891/0889-8391.32.4.223

Springer, K. S., Levy, H. C., and Tolin, D. F. (2018). Remission in CBT for adult anxiety disorders: A meta-analysis. *Clin. Psychol. Rev*. 61, 1-8. doi:10.1016/j.cpr.2018.03.002

Stain, H. J., Mawn, L., Common, S., Pilton, M., and Thompson, A. (2019). Research and practice for ultra-high risk for psychosis: A national survey of early intervention in psychosis services in England. *Early Interv Psychia*, 13, 47-52. doi:10.1111/eip.12443

Stevens, M. W. R., King, D. L., Dorstyn, D., and Delfabbro, P. H. (2019). Cognitive–behavioral therapy for Internet gaming disorder: A systematic review and meta-analysis. *Clin. Psychol. Psychother.* 26, 191-203. doi:10.1002/cpp.2341

Sun, H., Huang, H., Ji, S., Chen, X., Xu, Y., Zhu, F., et al. (2019). The Efficacy of Cognitive Behavioral Therapy to Treat Depression and Anxiety and Improve Quality of Life Among Early-Stage Breast Cancer Patients. *Integr. Cancer Ther*. 18. doi:10.1177/1534735419829573

Tang, W. X., Zhang, L. F., Ai, Y. Q., & Li, Z. S. (2018). Efficacy of Internet-delivered cognitive-behavioral therapy for the management of chronic pain in children and adolescents: A systematic review and meta-analysis. *Medicine* (United States), 97. doi:10.1097/MD.0000000000012061

Thabrew, H., Stasiak, K., Hetrick, S. E., Donkin, L., Huss, J. H., Highlander, A., et al. (2018). Psychological therapies for anxiety and depression in children and adolescents with long-term physical conditions. *Cochrane Database Syst. Rev.* 2018. doi:10.1002/14651858.CD012488.pub2

Thabrew, H., Stasiak, K., Hetrick, S. E., Wong, S., Huss, J. H., & Merry, S. N. (2018). E-Health interventions for anxiety and depression in children and adolescents with long-term physical conditions. *Cochrane Database Syst. Rev*. 2018. doi:10.1002/14651858.CD012489.pub2

Walczak, M., Ollendick, T., Ryan, S., & Esbjørn, B. H. (2018). Does comorbidity predict poorer treatment outcome in pediatric anxiety disorders? An updated 10-year review. *Clin. Psychol. Rev*. 60, 45-61. doi:10.1016/j.cpr.2017.12.005

Wang, L., Chang, Y., Kennedy, S. A., Hong, P. J., Chow, N., Couban, R. J., et al. (2018). Perioperative psychotherapy for persistent post-surgical pain and physical impairment: a meta-analysis of randomised trials. *Br. J. Anaesth.* 120, 1304-1314. doi:10.1016/j.bja.2017.10.026

Wang, S. B., Wang, Y. Y., Zhang, Q. E., Wu, S. L., Ng, C. H., Ungvari, G. S., et al. (2018). Cognitive behavioral therapy for post-stroke depression: A meta-analysis. *J. Affect. Disord*. 235, 589-596. doi:10.1016/j.jad.2018.04.011

Werner-Seidler, A., Johnston, L., and Christensen, H. (2018). Digitally-delivered cognitive-behavioural therapy for youth insomnia: A systematic review. *Internet Interv*. 11, 71-78. doi:10.1016/j.invent.2018.01.007

Ye, M., Du, K., Zhou, J., Zhou, Q., Shou, M., Hu, B., et al. (2018). A meta-analysis of the efficacy of cognitive behavior therapy on quality of life and psychological health of breast cancer survivors and patients. *Psychooncology*. 27, 1695-1703. doi:10.1002/pon.4687

Zaboski, B. A., and Storch, E. A. (2018). Comorbid autism spectrum disorder and anxiety disorders: A brief review. *Future Neurology*. 13, 31-37. doi:10.2217/fnl-2017-0030

Zhang, A., Borhneimer, L. A., Weaver, A., Franklin, C., Hai, A. H., Guz, S., et al. (2019). Cognitive behavioral therapy for primary care depression and anxiety: a secondary meta-analytic review using robust variance estimation in meta-regression. *J. Behav. Med.* doi:10.1007/s10865-019-00046-z

Zhang, A., Franklin, C., Jing, S., Bornheimer, L. A., Hai, A. H., Himle, J. A. et al. (2019). The effectiveness of four empirically supported psychotherapies for primary care depression and anxiety: A systematic review and meta-analysis. *J. Affect. Disord.* 245, 1168-1186. doi:10.1016/j.jad.2018.12.008

Zhou, X., Zhang, Y., Furukawa, T. A., Cuijpers, P., Pu, J., Weisz, J. R., et al. (2019). Different Types and Acceptability of Psychotherapies for Acute Anxiety Disorders in Children and Adolescents: A Network Meta-analysis. *JAMA Psychiatry.* 76, 41-50. doi:10.1001/jamapsychiatry.2018.3070
